# Usability of Mobile Solutions Intended for Diagnostic Images—A Systematic Review

**DOI:** 10.3390/healthcare10102040

**Published:** 2022-10-15

**Authors:** Jakub Kufel, Katarzyna Bargieł, Maciej Koźlik, Łukasz Czogalik, Piotr Dudek, Aleksander Jaworski, Mikołaj Magiera, Wiktoria Bartnikowska, Maciej Cebula, Zbigniew Nawrat, Katarzyna Gruszczyńska

**Affiliations:** 1 Department of Biophysics, Faculty of Medical Sciences in Zabrze, Medical University of Silesia, 41-808 Zabrze, Poland; 2 Faculty of Medical Sciences in Katowice, Medical University of Silesia, 40-752 Katowice, Poland; 3 Division of Cardiology and Structural Heart Disease, Faculty of Medical Sciences in Katowice, Medical University of Silesia, 40-055 Katowice, Poland; 4 Professor Zbigniew Religa Student Scientific Association at the Department of Biophysic, Faculty of Medical Sciences in Zabrze, Medical University of Silesia, 41-808 Zabrze, Poland; 5 Department of Radiology and Nuclear Medicine, Faculty of Medical Sciences in Katowice, Medical University of Silesia, 40-752 Katowice, Poland; 6 Foundation of Cardiac Surgery Development, 41-800 Zabrze, Poland

**Keywords:** mobile devices in radiology, mobile applications in radiology, mobile work with radiological images

## Abstract

Despite the growing popularity of mobile devices, they still have not found widespread use in medicine. This is due to the procedures in a given place, differences in the availability of mobile devices between individual institutions or lack of appropriate legal regulations and accreditation by relevant institutions. Numerous studies have been conducted and compared the usability of mobile solutions designed for diagnostic images evaluation on various mobile devices and applications with classic stationary descriptive stations. This study is an attempt to compare the usefulness of currently available mobile applications which are used in the medical industry, focusing on imaging diagnostics. As a consequence of the healthcare sector’s diversity, it is also not possible to design a universal mobile application, which results in a multitude of software available on the market and makes it difficult to reliably compile and compare studies included in this systematic review. Despite these differences, it was possible to identify both positive and negative features of portable methods analyzing radiological images. The mobile application of the golden mean in hospital infrastructure should be widely available, with convenient and simple usage. Our future research will focus on development in the use of mobile devices and applications in the medical sector.

## 1. Introduction

The first attempts to use mobile devices to display images in the Digital Imaging and Communications in Medicine (DICOM) standard were described in 2003. At that time, a Compaq iPaq Pocket PC was used, equipped with a mobile application based on a hypertext transfer protocol (HTTP) server called “Cyclops PDA DICOM Editor”. This solution enabled remote access to patient data and, most of all, visualization of diagnostic images, such as computer tomography (CT), ultrasonography (US) and magnetic resonance (MRI) [1].

On 7 June 2011, the World Health Organization (WHO) issued a statement concerning technical solutions, pointing to mobile devices as a potential remedy to the problem of distance medical consultation [2]. The development of IT technology, digitization, the Internet and the improvement of network infrastructure have established methods of transmitting radiological images between hospitals [3].

Despite technological progress and the continuous increase in popularity of mobile devices such as tablets or smartphones, they are not widely used in diagnostic imaging as its indispensable element yet. There are many mobile applications which are designed to display and work with radiological images; however, many of them are referred to as “not for diagnostic use” and only some of them are used clinically [4]. Nevertheless, mobile devices have become the key means of communication among medical staff in recent years. In a survey that was part of a study by Nerminathan et al. in 2016, 109 doctors indicated that 91% have smartphones, and 88% declared they often use mobile devices in clinical work [5]. What is more, the vast majority of clinicians (85.5%) declared the willingness and need to introduce mobile devices in medical practice throughout the country and 91.1% confirmed that they provide support in everyday work [6].

The purpose of mobile devices is to share medical data—they are therefore used for viewing and transmitting, often over long distances, radiological images which simplify consultation between clinicians. Their computing power allows them to transfer, manipulate and work with radiological examinations. However, the European Society of Radiology (ESR) does not recommend mobile devices for the primary interpretation, emphasizing that they can be successfully used to obtain additional opinions or to work at the patient’s bedside [7].

In emergency situations, quick access to imaging tests is crucial for the treatment process. Therefore, there is a need to create solutions which enable displaying and working with imaging examinations with the use of easily accessible mobile devices. Some studies report effective use of applications in order to improve diagnostic and decision-making processes, for instance in the conditions of the operating theatre [8]. Cewe et al. also showed that the necessary time to conduct a remote radiological consultation with the use of mobile devices compared to traditional description stations using the Picture Archiving and Communication System (PACS) is much shorter [9].

A systematic review of scientific reports was carried out with the use of publicly available medical databases, i.e., Pubmed, Online Wiley Library, Web of Science and Scopus in order to check the usefulness of mobile solutions intended for work on radiological research.

## 2. Materials and Methods

In order to ensure the transparency and credibility of this study, the systematic review was conducted in accordance with the PRISMA 2020 Statement guidelines and PRISMA 2020 Checklist (File S1: PRISMA 2020 Checklist) [10].

### 2.1. Search Strategy and Selection Criteria

Articles for this review were collected using literature from the public medical databases of Pubmed, Online Wiley Library, Web of Science and Scopus. MeSH terms “mobile dicom viewer” OR “dicom viewer phone” OR “dicom smartphone viewer” OR “dicom tablet viewer” OR “mobile dicom reader” OR “dicom phone reader” OR “dicom tablet reader” OR “dicom smartphone reader” were entered in each of the previously mentioned databases. Searching for articles was restricted by filters: articles only in English, and only articles with an abstract. A total of 84 studies were obtained, 26 of which were automatically removed due to duplications in other databases. The entire article database is available in supplements to this article (File S2: Articles database). The review was conducted in February 2022.

### 2.2. Data Extraction and Quality Assessment

**Figure 1:**
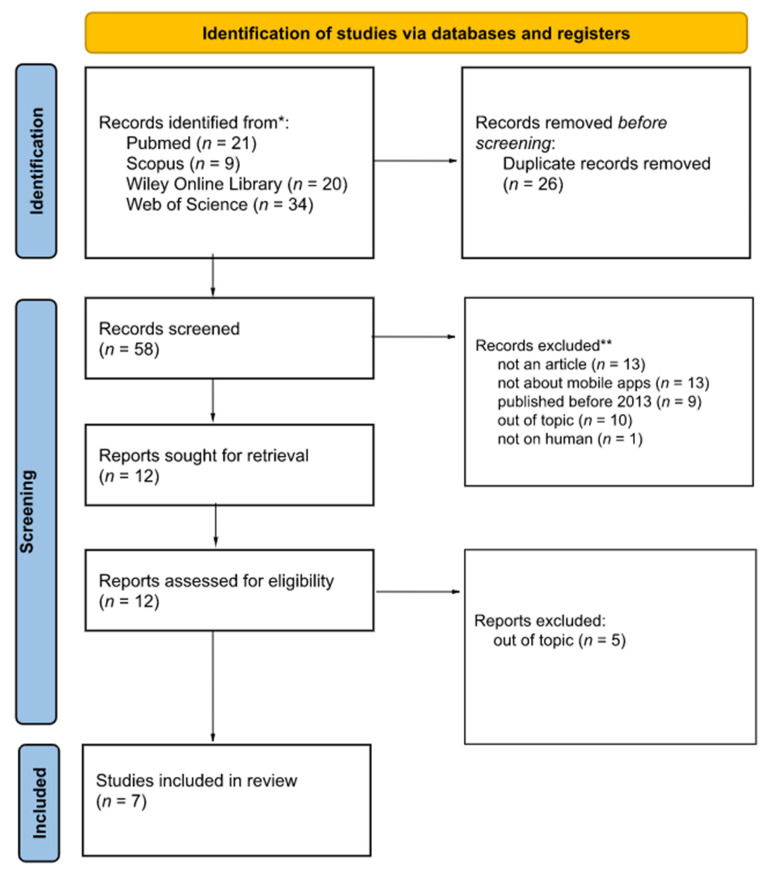
PRISMA 2020 flow diagram.

Selected articles were exported from scientific databases and then imported to the Rayyan Qatar Computing Research Institute [11]. Each of them was independently evaluated by two reviewers. The articles were assessed for suitability in this study (whether the article concerns the topic of this systematic review, whether the article compares the quality of the DICOM image review on mobile devices with quality of image review on descriptive stations, whether the article has free access, whether the article concerns patients, if the article was published in 2013 or later, and whether the article has enough data for analysis). Articles published before 2013 do not provide up-to-date data and knowledge; therefore, they were not considered for this study. Taking into account the pace of technology development, the possibility of an application becoming obsolete is very high. A 10-year cut-off point was selected to ensure that the articles included in this review continue to provide useful and realistic information. A total of 58 articles were included in the beginning. Afterwards, 46 articles were excluded due to: the form of a study is other than an article (*n* = 13), there is lack of information on the use of mobile applications (*n* = 13), article is published before 2013 (*n* = 9), topic is not related to the subject of this work (*n* = 10), or the topic of work is not related to people (*n* = 1). Some articles could have been excluded due to more than one exclusion category; however, for the sake of simplicity of this review these papers were assigned to the group that was observed first. Cohen’s kappa was estimated at 0.76 (agreement in 91.7%), which is interpreted as agreement on fundamental issues between the authors [12]. Conflicts that arose after the articles were initially qualified for the study were resolved by a third independent researcher. In this way, 12 articles were received, of which, after careful analysis, the entire research team selected 7 important ones for this systematic review. The excluded articles were removed for being not compatible with the topic (*n* = 5). PRISMA 2020 flow diagram is presented below (Figure 1).

### 2.3. Detailed Inclusion Criteria

In order to qualify for this review, an article had to meet the following criteria: it had to include information about the type of application and its purpose, as well as the operating system on which it was running; it had to compare the quality of the image review on the mobile device and image review on the descriptive station; it had to have an appropriate amount of data which enabled the analysis. The requirements for the application described in the article were the ability to work on radiological images.

## 3. Results

Of the seven studies that were included in this systematic review, six of them were prospective, and one was retrospective. Basic information on the studies included in this review is presented in Table 1. All articles were published in the period from the beginning of 2013 to the end of 2021. Studies present performance of mobile applications and browsers operating on descriptive diagnostic stations.

The following applications were used for image analysis in DICOM format on descriptive stations: OsiriX [13,15], eFilm [14] and CB-Works [16]. On mobile devices it was OsiriX mobile [13,14,15], mRay [17,19], Bone Ninja [18] or OsiriX via Pocket Cloud [16].

### 3.1. Aims of Included Articles

Choudhri et al. undertook the assessment of the diagnostic capabilities of a mobile browser in the interpretation of abdominal computed tomography examinations. The studies were conducted to determine the possibility of initial identification and classification of dissecting aneurysm, aortic rupture, intramural hematoma, determining the dimensions of the aorta, identifying mediastinal hematoma, aortic arch variants and pulmonary pathologies [13].

De Maio et al. evaluated and compared the diagnostic performance of handheld mobile devices to a conventional PACS workstation for radiological image description using knee MRI scans. Interpretation sensitivity and specificity of test results on both types of devices were also compared [14].

Abboud et al. aimed to compare the reproducibility of detecting pulmonary tuberculosis lesions on an iPad 2 mobile device with Osirix HD software to a classic description station with a liquid crystal monitor [15].

Carrasco et al. compared the diagnostic accuracy of a PACS, a consumer-grade monitor, a laptop and a tablet when performing linear height and width measurements for specific dental implant placement sites (in the back of the maxilla and mandible). The visualization quality of the related anatomical structures was also analyzed, as well as the assessment of trabecular bone and its correct structure while maintaining the highest image quality. The aim was to answer the question which of the mentioned above devices will be the most beneficial in clinical work. A total of 32 computed tomography examinations were subjected to such evaluation. Additionally, the subjective feelings of PACS and iPad users were assessed on the basis of a short questionnaire [16].

The aim of the work by Vetter et al. was to answer whether tablets can speed up the process of searching for radiological images—entire studies, series of photos and individual DICOMs. The study was conducted among physicians who were asked to give a second opinion about the imaging test results of trauma center patients. On the basis of a short, proprietary questionnaire, the accessibility to images and the frequency of usage in everyday work were assessed [17].

Whitaker et al. assessed the accuracy of the limb deformation measurements with the Bone Ninja app compared to PACS. Intra- and inter-observer variability among different orthopedic practitioners were also determined [18].

In a study by Brehm et al. mRay performance was assessed on two mobile devices and compared to a GE PACS workstation. Authors also evaluated whether software quality is sufficient to assess computed tomography of patients with suspected stroke [19].

### 3.2. Methodology of Included Articles

In a study by Choudhri et al., an iPhone 3G is equipped with a display with a resolution of 420 × 380 pixels with 163 pixels per inch. Assessment of DICOM computed tomography datasets was performed on 16 or 64 slice helical scanners after intravenous contrast administration. The image resolution was 512 × 512 pixels. Image data were first transferred to a MacBook Pro computer, then loaded into the OsiriX DICOM viewer and anonymized. Later, the images were transferred to an iPhone 3G device. Image analysis was performed by three radiologists, previously trained to use both the device and software. They assessed presence of aortic dissection, intramural hematoma, pseudoaneurysm, aortic transection or active extravasation. Diagnoses made on the mobile device were compared to the assessments made on the PACS workstation by radiologists specializing in vascular imaging [13].

De Maio et al. compared magnetic resonance imaging (MRI) examinations of the knee joints on a mobile device and a standard PACS station. The mobile device used in the study was an iPhone 3GS, iOS 4.0 with a screen diagonal of 8.9 cm and a display with a resolution of 320 × 480 pixels. Software used on the mobile device was OsiriX DICOM viewer, version 1.1.4. A second device was a Hewlett-Packard Z800 workstation, equipped with the Windows XP pro 2002 service pack 3, connected to an HP LP3065 monitor with a screen diagonal of 76.2 cm and a display with a resolution of 2560 × 1600 pixels. The eFilm Workstation 3.0 program was used to view images on the workstation. The images for the examination were obtained using a 1.5-T MRI machine, Signa Excite, software version 12.0, with a multichannel coil dedicated to the knee joint. Examinations were performed according to the standard knee MRI protocol. The layer was 4 mm thick and the field of view was 14 × 14 cm. The examinations were assessed by two radiologists with longstanding experience in diagnosing diseases of the musculoskeletal system on MRI images. Researchers worked independently, in a blinded and randomized manner. Examinations were displayed at random. The first researcher assessed all 50 images. The second researcher, in order to ensure credibility, described 25 randomly selected studies from the entire pool of studies analyzed. To minimize the risk of distorting results due to researchers remembering the content of previously analyzed examinations, the time interval between the interpretation of the same test on the mobile device and the PACS station was at least two months. A report of the examination description was written according to the standard formula used by the orthopedic surgeon as well as the time needed for the examination description was measured, rounded to the nearest minute [14].

Abboud et al. compared two devices in their study: an iPad2 with screen diagonal 9.7” with a resolution of 1024 × 768 pixels, maximum brightness 410 cd/m^2^ and contrast ratio 962:1 and as a comparison monitor, an IMac LCD with screen diagonal 17”, 2560 × 768 pixels resolution, maximum brightness 375 cd/m^2^ and default contrast ratio 1000: 1. The iMac’s DICOM image viewer was OsiriX Dicom and the iPad 2’s was OsiriX HD. The study used a collection of 240 chest X-rays from the tuberculosis screening program. Of these 240, 200 cases were originally reported negative, and 40 were positive. All studies were anonymized and prepared for interpretation in random order. Examinations were evaluated by a group of five radiologists whose task was to assess binary (positive or negative) presence of radiological features characteristic for tuberculosis. The first device to evaluate diagnostic images was selected randomly for each researcher. After at least 1 week researchers assessed images on the second device. The readings were taken in similar lighting conditions in the same room [15].

Carrasco et al. used the following devices and software as readers for CT scans: PACS medical grade LCD monitor “Planar PX212M”; Consumer grade LCD monitor “HP Compaq LA2205wg”, CB-Works software; MacBook Pro 13, OsiriX DICOM reader software; “IPad-4”, OsiriX DICOM reader software. Operators were to evaluate 32 CBCT (Cone Beam Computed Tomography) scans made with the CBCT Hitachi CB MercuRay using the standard departmental protocol (120 KV and 15 mA). Prior to evaluation, the images were anonymized and randomly numbered. Using an external storage device, DICOM data were transferred to the target device used in the study: Hewlett-Packard xw8200 Microsoft Windows XP, version 2002, Pack 3, (on a PACS workstation); HP Compaq 8200 Elite Microsoft Windows XP professional, version 2002, Pack 3; MacBook Pro, Mac OS X, version 10.8.3. The data were then imported into software (CB-Works, OsiriX). The PACS monitor was used as the gold standard. The iPad 4 was connected to a MacBook via a wireless network with the help of an application supporting a remote desktop (Pocket Cloud). In addition, the iPad and the stylus were secured with a clear plastic film to simulate the sterility conditions of the operating theater. The images were viewed and assessed by operators under standardized light and sound conditions. To simulate the conditions of a dental practice, images displayed on a tablet equipped with the OsiriX program were viewed in a room with fluorescent lighting. The operators used the tools provided by the programs including magnification, contrast brightness, cross-section cutting tools and length measurement. The region of interest (ROI) was the edentulous areas of the mandible and maxilla around the molars and premolars. The diagnostic quality of the devices was checked by the study participants by performing quantitative and qualitative measurements, other qualitative measures and the device resolution essential to determine pathology [16].

The Ortho Mobile study by Vetter et al. focuses on the usage of mobile devices and specialized software by orthopedists for viewing radiological images in clinical settings. An iPad mini 2 tablet was used due to a screen size sufficient to evaluate the imaging tests, but small enough to fit in the apron pocket. The software was selected in terms of its ability to work offline. The function of exchanging messages with the help of messenger was also noted. The selected application—mRay—was created for reading and analyzing images on mobile devices in the DICOM format. The software consists of the actual application (client component) and the application server. The task of the application server is to retrieve data from the PACS database, encrypt and compress. Then, these data are sent to the mobile device—their further reading after downloading can take place without any network connection. The mRay application is a CE-certified medical tool. The communication platform, which is part of the application, enables (in addition to the exchange of text and audio messages) the sharing of DICOM images or their fragments between users. Doctors participating in the study were trained in using the software. The results of operating the device were recorded by completing an online questionnaire daily [17].

Whitaker et al. compared the use and accuracy of PACS and Bone Ninja mobile applications in limb deformity measurements. Four evaluators with different levels of experience (an attending orthopaedic surgeon, a senior orthopaedic resident, a junior orthopaedic resident, and an orthopaedic physician assistant) measured each image (48 limb images of 24 patients) on four individual occasions (twice with the Bone Ninja application and twice on PACS) with at least one week interval between measurements and made use of PACS and Bone Ninja alternately. Each of the evaluators received detailed information on how to perform measurements on both devices. The time of their assessment was measured when they last carried out an evaluation. Measurements taken for each image included right and left limb total length (LL), lateral distal femoral angle (LDFA), and medial proximal tibia angle (MPTA). All images were calibrated with a 2.54 cm calibration ball. The PACS measurements were completed on a 10.5 × 13-inch monitor, while the mobile device was a 4th generation iPad with a 9.7-inch retina display. Additionally, the satisfaction of the evaluators was assessed by asking which device they would prefer to use in the future for deformation measurements [18].

Brehm et al. evaluated the diagnostic quality of the mRay image viewing software and a classic radiology workstation. One experienced neuroradiologist (>5 years of experience) and one resident (>1 year of experience) appraised the anonymized cases of 50 patients (there were multiple images of each of the patients) separately on two handheld devices (iPhone 7 Plus, MED-TAB) equipped with mRay Software and on a GE PACS workstation. The iPhone 7 plus had 128 GB of flash memory and a 5.5-inch diagonal screen with 1920 × 1080 pixels (luminance = 625 cd/m^2^). The MED-TAB had 16 GB of flash memory and a 13.3-inch diagonal screen with 1920 × 1080 pixels (luminance > 250 cd/m^2^). They were DICOM Part 14 greyscale standard display function-certified. GE PACS was connected to two medical-grad 21-inch liquid crystal displays (RX250, EIZO), both with a resolution of 1000 × 1600 pixels and a luminance of 400 cd/m^2^. To minimize the likelihood of recalling the image, each case was reviewed on each of the three devices with at least a 12-week interval. Both reviewers were asked to view the images in a location with ambient lighting below 100 lux. Both physicians were also asked to evaluate the diagnostic quality of all three devices on a five-point ordinal scale regarding detection of large-vessel occlusion (LVO), intracranial hemorrhage (ICH), early ischemic signs and overall safety of the diagnosis [19].

### 3.3. Results of Included Articles

In a study by Choudhri et al., aortic pathology was correctly identified on mobile devices (9/9) in all abnormal studies out of 15 reviewed studies. Additionally, all non-pathologic tests (6/6) were classified correctly. Other abnormalities (mediastinal hematomas, pneumothorax and aortic arch involvement) were also aptly observed. Abnormal aortic arch types (bovine-type arch, left vertebral arteries originating between the left common carotid and left subclavian) were also observed. One researcher did not recognize one of these aberrations once. In patients with aortic pathologies, the diameters of these pathologies were measured. The following results were obtained and then compared with the PACS results: the maximum diameter of the pathology was 42.4 ± 10.5 mm (range 32–64 mm) on a mobile device and 40.2 ± 8.5 mm (range 28–56 mm) on a dedicated PACS station. Diameters of the descending aorta were also measured at a site not affected by pathology for comparison. The following results were obtained: 25.6 ± 4.7 mm (range 19–33 mm) on a mobile device and 24.5 ± 4.2 mm (range 18–31 mm) on a PACS workstation (*p* = 0.03). According to the authors, the diagnosis made on the basis of DICOM images on the mobile device does not differ from the assessment made after the review of the same images on the PACS workstation. The authors also concluded that a larger screen could be a “useful tool” [13].

De Maio et al. used image examinations of all 50 patients. These patients underwent MRI of the knee, followed by knee arthroscopy in the period from 1 January 2009 to December 31, 2009. In 50% of cases, the most common indication was damage to the meniscus, and in 24%, damage to the anterior cruciate ligament. The iPhone evaluations showed high specificity (from 74% for cartilage to 100% for PCL) and sensitivity (from 77% for the lateral meniscus to 100% for ACL). Equally high sensitivity and specificity were demonstrated by observations made on standard workstations (specificity from 84% for cartilage to 100% for PCL, sensitivity from 82% for the lateral meniscus to 100% for ACL). The difference between the two types of reading was the amount of time needed to make them (mean difference between the two types of reading was 3.98 min). Longer time was necessary for assessment of images on the iPhone [14].

Abboud et al., during screening for tuberculosis in 240 patients, compared the LCD monitor (27′’ screen diagonal) with the iPad display (9.7′’ screen diagonal). Cohen’s Kapp analysis for five researchers and two displays showed that it is extremely good (>0.8) or very good (>0.79) depending on the professional experience of the researcher. All researchers agreed that the evaluation of radiographic images on the smaller screen of the mobile device was slower than assessment on the LCD monitor. However, this only affected the comfort of work and not the interpretation of the image itself. The results of the study clearly confirm that tablets can be used for radiological diagnosis of tuberculosis with the same effect as consumer LCD monitors (generalized Cohen’s κ in many assessments was calculated as 0.865 (z = 15.7) for the iPad and 0.817 (z = 14.8) for the display LCD). Compliance in making the diagnosis on LCD monitors and tablets reached 90%, and for cases classified as negative was higher (94%) than for those classified as positive. However, the authors do not recommend the use of mobile devices for routine diagnostics. They note that their research does not allow for the possibility of detecting other lung diseases, especially those characterized by small, hardly noticeable changes [15].

Carrasco et al. obtained results clearly showing the high compliance of the measurements for all used devices, including the tablet. All *p*-values by the Greenhouse–Geisser test were greater than 0.05, i.e., 0.127; 0.195; 0.121; 0.093, indicating no significant difference between monitors for each of the measurements taken on 32 patients. ICC values showed high reliability. The measurements taken for the second time were even more compliant than the first ones and technical parameters of all tested monitors allowed obtaining consistent results. The authors indicate that the diagnostic quality of the tests, and the visualization of specific points and the cancellous bone were identical in the evaluation process on both devices during both assessing sessions. The exception is the measurement of the inferior alveolar nerve canal (IAC) with a difference of one. For the IAC measurements, a Kappa was also calculated with a value of 0.9130 and a corresponding *p*-value (<0.0001). These results show good intraoperator agreement. Additionally, the authors of the study asked clinicians to complete a short questionnaire assessing their subjective experience of using different devices and programs. The results of the survey clearly indicate the almost complete absence of subjective differences in the iPad’s and PACS’ quality of use, pointing to the mobility of the tablet as its greatest advantage. On a scale of 1 to 5, in the question about mobility, iPad received 5 points, and PACS only 1 point. In terms of comfort of use, speed of viewing images and ease of manipulation, the iPad scored 4 points and PACS 5 points in each of the above-mentioned survey questions. According to the authors, the iPad is additionally distinguished by its competitive price, mobility and the possibility of intraoperative use, minimizing the risk of infections for the patient which provides additional support and facilitates the work of implantologists [16].

Vetter et al. showed that viewing radiological images on tablets accounted for, on average, less than 30% of daily analyzed images. Additionally, mobile devices were used 1.1 times a day (on average) for bedside demonstration. Based on the measurements of the access time to the diagnostic images, it was also found that the mobile device is more than twice as fast as a desktop computer (1 min and 2.2 min, respectively). The mean difference in these measurements (estimated using the linear mixed effects model) was 1.1 min in favor of mobile device access. The tablet was used in about 29% of the total number of 425 requests for consultation, which resulted in an average of 1.7 per day. Only in 0.2% of cases, it was necessary to reanalyze a given study on a desktop computer due to insufficient visibility on a mobile device. The authors also received complaints about connecting to the WiFi network that made their work difficult, but it should be noted that the mobile device was used throughout the hospital, both in the ward, in the operating room and in the emergency room [17].

In the article by Whitaker et al., all four clinicians who measured LL, LDFA, and MPTA of 24 patients on both devices had excellent agreement of correlation both within the observer (intra-observer correlation) and between researchers, based on Cohen’s kappa. There were no statistical differences in the leg length discrepancy (LLD), MPTA or LDFA measurements between Bone Ninja and PACS (*p* = 0.96, 0.87 and 0.97, respectively). The intra-observer, intra-class correlation coefficient (ICC) for the measurements was similar when using the Bone Ninja and PACS applications (0.83, 0.89 and 0.96 vs. 0.96, 0.93 and 0.95, respectively). The ICC between the observers was also similar between the mobile application and the classic descriptive station (0.95, 0.96 and 0.99 vs. 0.99, 0.98 and 0.98, respectively). Length measurements of the right and left lower limbs were compared in terms of their accuracy—there was no difference in accuracy between them, neither with the PACS nor with the Bone Ninja (*p* = 0.526). Moreover, in the opinion of doctors assessing radiographic images, the Bone Ninja mobile application was more pleasant and faster to use than the classic descriptive station, as shown by the results of the time measurements (an average of 3 min and 43 s per image for Bone Ninja and 4 min. 51 sec. for PACS) [18].

In a study by Brehm et al., both senior neuroradiologist and the resident correctly identified all LVOs, intracranial tumors and ICHs on both mobile devices and PACS. In the case of an experienced clinician, the selection of the LVO (large vessels occlusion) location differed three times. He diagnosed 12 severe stenoses (3 VA (arteria vertebralis) and 9 ICA (arteria carotis interna)) and the resident diagnosed 14 severe stenoses (4 VA and 10 ICA). In the CCT (cranial computer tomography) and CBV (cerebral blood volume) ASPECTS (Alberta stroke program early computer tomography score) assessment, both investigators received a median score of 10 on all three devices and there was no statistically significant difference between the assessments of both specialists. The sensitivity of CBV/CBF (cerebral blood flow)-mismatch detection for a senior neuroradiologist was 84.2% for MED-TAB and 88.2% for iPhone 7 plus, while for a resident it was 85.0% and 85.0%. The corresponding specificity was 91.3% for MED-TAB and 90.9% for iPhone 7 Plus in the case of a senior neuroradiologist, or respectively 84.2% and 83.3% as assessed by the resident. Both intracranial tumors visualized in patients were correctly identified on all three devices by both doctors. Both clinicians with high certainty ruled out intracranial bleeding (ICH) on both mobile devices and rated their diagnostic value as “adequate” in 97.8% of the cases and as “excellent” in 82.4% of the cases. According to an experienced neuroradiologist, early symptoms of cerebral ischemia could be diagnosed in 94% of cases on both mobile devices and in 98% of cases in GE PACS, but the difference is not statistically significant (*p* = 0.946 and *p* = 0.112). The resident recognized them with GE PACS in 92% of cases, MED-TAB in 96% (*p* = 0.699), and in 95% on iPhone 7 plus (*p* = 0.893). Overall, the senior doctor rated both mobile devices and the GE PACS system as sufficient for diagnosis in all cases. The resident judged MED-TAB as sufficient for diagnosis in 96%, iPhone 7 plus in 94%, and GE PACS in 98%, which are not statistically significant differences (*p* = 0.181 and *p* = 0.956) [19].

## 4. Discussion

All articles included in this review point out how important it is for mobile applications cooperating with radiological examinations to be easy to use and have a user-friendly interface. At the same time, they should not be inferior to devices traditionally used during the diagnostic process in terms of the variety of possible manipulations of a given image [20]. Shih et al. stated the interface plays an extremely important role in the use of a given device while influencing performance, discoverability and frequency of use [21]. In addition, Whitaker et al. described that taking the measurements of anatomical structures on a mobile device (using the Bone Ninja application) was faster compared to PACS [18]. Vetter et al. emphasized the time required for opening an image on a mobile device (using mRay—1.1 min) was notably shorter compared to the PACS workstation (2.2 min). This example proves the superiority of mobile systems in terms of time needed to open an application—utilizing tablets significantly abridges workflow of physicians. Images can undergo review directly after being sent to the mobile device; however, in order to use PACS, the physician must first get to the place where the PACS is located [17]. In addition, Jenei et al. mention that the radiological images on mobile devices can be accessed everywhere, which is a faster alternative compared to the PACS workstation and is especially useful in urgent cases [22]. 

Diagnosis with the use of DICOM images on a mobile device can be made with a good concordance compared to PACS description workstations [23]. McEntee et al. looked at the efficiency of using an iPad to evaluate chest X-rays to detect lung nodules. The reference standard was the LCD display used in PACS stations. The results of the 30 images’ interpretation by eight radiology specialists showed comparable effectiveness of both methods (for iPad, ROC = 0.76; JAFROC FOM = 0.66. For LCD, ROC = 0.79; JAFROC FOM = 0.74) [24].

An additional advantage is the use of mobile devices in the aseptic conditions of the operating theater—smaller dimensions of the device facilitate the maintenance of sterility, which may be of particular importance in interventional dentistry, orthopedic surgery or maxillofacial surgery. This is indicated by Carrasco et al., who tried to simulate the operating room environment and maintain sterilization protocols. They used an iPad while covering the device with a transparent plastic film. Measurements were performed with a stylus placed in a closed plastic bag, particularly designed for devices with a touch screen, thus obtaining conditions which minimize the risk of infection in the patient [17]. The effectiveness of such a solution is also confirmed by Hammon et al.—securing the tablet with a tight plastic foil bag effectively reduces the degree of microbiological contamination of the device in clinical conditions, simultaneously protecting users against the spread of potentially pathogenic bacteria. Moreover, this protection does not significantly affect the comfort of use. The above-mentioned method provides an aseptic possibility for the implementation of mobile devices in procedures that require sterility, as disinfection of these devices with disinfectants is only possible to a small extent and may reduce their service life [25].

When deciding to use mobile devices, users should take into account the possibilities of their cooperation with the IT environment in a given facility. Therefore, it is extremely important not only to adapt them to work with many browsers and/or software programs, but also to pay special attention to the data transfer rate [13]. It may also be convenient to use DICOM’s web-based browsers, which, unlike traditional software applications, do not need to be downloaded or installed. However, this may be inconvenient in some circumstances. What is more, not all browsers may support a given application. Depending on the technical capabilities of the equipment, the average transfer time of a single chest CT scan may take about 5 min via wireless LAN [13]. In a study by Kammerer et al. researchers (using a mobile device) managed to obtain the following data transfer rates, i.e., 86 s for a thoraco-abdominal CT, 63.67 s for a neck CT and 20.78 s for a head CT [26]. In turn, Choudhri et al. showed that sending one CT image takes 0.6 s using Wi-Fi with a speed of 1.919 MB/s [27]. Vetter et al. were the only ones who tried to estimate the time needed to obtain a set of images for evaluation on a tablet and PACS station, obtaining information based on questionnaires filled in by clinicians. Average time of opening the image of a particular patient was 2.2 min on a standard computer and about 1 min when using a tablet, which indicates faster availability for testing on a mobile device [17]. In large clinical centers, the infrastructure of which consists usually of several buildings, an additional problem is the lack of access to Wi-Fi in some places. However, this can be solved by using an offline software [17]. When using mobile devices, users should take into account the delay in data transmission as well, which can be caused by a too weak Wi-Fi signal. The quality and availability of the data transfer infrastructure should be carefully verified in the conditions of relatively high-developed technologies, such as those offered by large municipal medical centers in a country with large technological resources [28]. When introducing mobile technologies in a given medical facility, users should take into account all limitations affecting possible radiological consultations, including the data transmission speed [14]. Filip et al., at the time of publication of their article, achieved a maximum data transfer speed of 512 kb/s, which allowed for sufficiently fast and reliable image data transmission via the mobile network. It was enough for a precise and accurate remote diagnosis of a patient. However, the authors point out that the data transmission speed will most likely increase in the future, which will improve the quality of image data sent by mobile devices [29].

In addition to physical and technological conditions, it is also necessary to take into account the human factor—the variety of devices and operating systems may be difficult for some personnel to operate. An additional difficulty may be the touch screens present in mobile devices—the way they are operated makes it difficult to keep the screen clean, which is important in enabling diagnosticians to see all the important details of a given examination [7]. This directly translates into the effectiveness of using mobile devices in diagnostics, which contradicts one of the flagship advantages of this method—acceleration of the diagnostic and therapeutic process [30].

Updating legal regulations is just as important as technology development or personnel education. The first mobile application approved in 2011 by the FDA was MobileMIM, while using an iPad for the first time for diagnostic purposes [31,32]. The agency points out that it will only standardize mobile applications that “could potentially have an impact on patient safety, such as radiation dose calculators” and “turn a mobile device into a regulated medical device, such as one accessing software requires Releasable 510 (k) Database approval” [33]. It is worth noting that although the FDA is an important determinant of standards in the technological solutions sector, applications allowed for clinical diagnostics must be approved by the relevant authorities of the country where they will be included in the diagnostic and therapeutic process or by another parent organization (e.g., in the case of the European Union).

Speaking of the constant availability of mobile devices, the most noteworthy thing is the fact that, thanks to their small size, you can always have them with you. Despite the fact that smaller screen surfaces in tablets and smartphones imply certain limitations (for example related to the number of simultaneously displayed tests or difficulties related to the operation of the application interface on a small screen), these devices positively distinguish themselves from traditional descriptive workstations [28]. In life-threatening emergencies, there may occur a situation where quick access to a PACS station is not possible. The use of a mobile device will enable an initial management decision with a patient, such as the preparation of the operating room and necessary medical staff at a time when surgeons can view DICOM images again at a dedicated descriptive workstation as well as ask radiologists for a second opinion who in a given moment may not be nearby or have no access to the research station. In emergency radiology, where time plays a key role, mobile devices are perfect for displaying examinations anytime and anywhere, outside the hospital infrastructure or for example by surgeons at the patient’s bedside or in the operating room. Mobile devices also work well in the hospital emergency department, where they allow for quick consultations between specialists, entire teams or when transferring patients to another facility [7]. According to the study by O’Connell et al., using mobile devices has a positive impact on the relationship between the radiologist, other team members and the patient, as well as brings radiologists closer to specialists in other branches of medicine [34].

## 5. Limitations of This Study

The main limitation of this systematic review was the very small number of studies included in this work due to the lack of articles consistent with the purpose of this review. The large variety of issues raised in the articles made it impossible to carry out an analysis and comparative analysis of individual articles. Despite the detailed selection of articles (containing a comparison of mobile devices using different DICOM browsers with PACS stations) for review, some authors in their research provided detailed technical data on the devices, browsers and applications they used, while other authors focused on the clinical usefulness of these devices, ignoring technical data, which makes comparative analysis of selected studies difficult.

## 6. Conclusions

In order for a given application to be used regularly, its operation must be simple and comfortable—one of the most important factors is the interface. The undoubted advantage of the mobile application is the lack of limitations as to the place of use, both in hospital conditions (also in the operating theater) and out of hospital. This feature is especially desirable in emergency situations. The devices should be properly secured, for instance with clear plastic film, so that they can be used in conditions requiring complete asepsis. The possibility of having a mobile device always with the user facilitates the consultation between the user and the second doctor. In order for mobile applications to be used at work by a physician, they must show a sufficiently high quality of analyzing radiological examinations (the most frequently mentioned factor was the screen size) and should contribute to the diagnosis and implementation of appropriate therapeutic procedures. The mobile devices must be compatible with IT systems installed in medical facilities, which should have an adequately efficient network infrastructure. One of the main problems can be a poor or scattered internet connection but this factor will probably improve in the future. There are still no relevant legal regulations that would clearly define the conditions for using the above-mentioned applications and devices in the diagnostic and therapeutic process.

## Figures and Tables

**Table 1:** Articles included in the systematic review.

Number	Authors	Title	Device	Application
[13]	Choudhri et al.	Diagnosis and treatment planning of acute aortic emergencies using a handheld DICOM viewer.	MacOs/iOs compatible	OsirX/OsirX moblie
[14]	De Maio et al.	Diagnostic accuracy of an iPhone DICOM viewer for the interpretation of magnetic resonance imaging of the knee.	Windows XP/iOs	eFilm Workstation/OsiriX mobile
[15]	Abboud et al.	TB or Not TB: interreader and intrareader variability in screening diagnosis on an iPad versus a traditional display.	MacOS/iOs compatible	OsiriX/OsiriX mobile
[16]	Carrasco et al.	Analyzing Dental Implant Sites From Cone Beam Computed Tomography Scans on a Tablet Computer: A Comparative Study Between iPad and 3 Display Systems.	MacOs; Windows XP/Pocket Cloud (remote desktop app)	CB Works/(OsiriX via Pocket Cloud)
[17]	Vetter et al.	Tablets for Image Review and Communication in Daily Routine of Orthopedic Surgeons—An Evaluation Study.	Apple iPad Mini 2 PACS (no specified data)	mRay
[18]	Whitaker et al.	Comparison of PACS and Bone Ninja mobile application for assessment of lower extremity limb length discrepancy and alignment.	Apple iPad 4 PACS (no specified data)	Bone Ninja
[19]	Brehm et al.	Image review on mobile devices for suspected stroke patients: Evaluation of the mRay software solution.	GE PACS/MED-TAB or iPhone 7 plus	mRay

## Data Availability

Search results are available from the authors.

## Supplementary Materials

The following supporting information can be downloaded at: https://www.mdpi.com/article/10.3390/healthcare10102040/s1, File S1: PRISMA 2020 Checklist; File S2: Articles database.
